# Progress in the Application of Single-Cell Sequencing in Neoadjuvant Therapy for Esophageal Cancer

**DOI:** 10.7759/cureus.89163

**Published:** 2025-07-31

**Authors:** Sang Houyi, Sun Zhiqiang, Wang Jianlin, Ni Xinchu, Luo Judong

**Affiliations:** 1 Department of Radiotherapy, The Affiliated Changzhou Second People's Hospital of Nanjing Medical University, Changzhou, CHN; 2 Department of Radiotherapy, Tongji Hospital, School of Medicine,Tongji University, Shanghai, CHN

**Keywords:** esophageal cancer, neoadjuvant therapy, sc-seq, tumor, tumor microenvironment (tme)

## Abstract

In recent years, with the continuous development of single-cell sequencing (sc-seq) technology, Sc-seq can conduct high-throughput and high-resolution transcriptome analysis at the single-cell level, revealing gene expression differences and molecular characteristics among individual cells. This provides more precise guidance for determining tumor molecular subtypes and formulating treatment strategies. The neoadjuvant therapy (NAT) for esophageal cancer, including chemotherapy, radiotherapy, molecular targeted therapy, immunotherapy, and comprehensive treatment, has achieved significant progress. However, there remain considerable challenges in enhancing the quality of life for esophageal cancer patients, extending their survival period, and reducing the rate of late-stage recurrence. This article explores the Sc-seq technology, delineating its advantages and disadvantages. It further examines the application progress of this technology in the tumor microenvironment (TME) of esophageal cancer, as well as its application for various neoadjuvant treatment regimens. The article offers a novel perspective on the effectiveness of NAT and provides a basis for the neoadjuvant treatment of esophageal tumors.

## Introduction and background

Esophageal cancer is the eighth most common cancer in the world. Among all tumors, it ranks seventh in incidence and sixth in mortality. Esophageal cancer is an invasive malignant tumor with insidious symptoms. It is often detected at the middle or advanced stage, resulting in poor overall prognosis, with an overall five-year survival rate of 15%-25% [1]. The main histological subtypes of esophageal cancer include esophageal squamous cell carcinoma (ESCC) and esophageal adenocarcinoma (EAC), and the tumor pathological type is closely related to the treatment plan [2]. Currently, surgical treatment is the primary approach for early-stage esophageal cancer. For locally advanced esophageal cancer, neoadjuvant therapy can effectively improve the efficacy and prognosis, bringing survival benefits to patients. However, there are significant differences in the NAT efficacy among different patients. Therefore, it is necessary to explore more accurate biomarkers for NAT treatment of esophageal cancer and the molecular mechanisms of treatment resistance [3,4]. Conventional sequencing, such as RNA-sequencing (RNA-seq), involves extracting the mixed RNA (bulk RNA) from tissues, organs, or a group of cells for sequencing, and the obtained data are the average of the transcriptomes of a group of cells. The specific information of individual cells in the cell population is often masked [5,6]. Therefore, single-cell sequencing (sc-seq) detects the gene expression level at the single-cell level, providing the possibility to study the complex mechanisms of the occurrence and development of esophageal cancer and tumor heterogeneity.

## Review

Overview of sc-seq sequencing technology

The applications of sc-seq technologies, such as single-cell transcriptomics, genomics, epigenomics, and multi-omics, have enabled in-depth analyses of individual cells at the levels of transcriptome, genome, and immunome. They each have their own advantages and disadvantages, listed in Table 1

**Table 1:** Common sc-seq methods and their advantages and disadvantages RNA-seq: RNA-sequencing; sc-seq: single-cell sequencing

Technology type	Specific method	Advantages	Disadvantages	Typical applications
Single-cell RNA-seq	Microwell-based	High single-cell capture efficiency enables the detection of alternative splicing and novel transcripts	Low throughput and high cost; requires high RNA input	Rare cell transcriptome analysis and novel transcript discovery
					Microfluidic platforms	High-throughput and low cost per cell	Only sequences transcript ends; sensitive to cell viability	Large-scale cell atlas construction and TME heterogeneity
	In situ sequencing	Resolve gene expression in TME and avoids gene expression changes from cell dissociation	Low throughput and technically complex	Spatial transcriptomics of embryonic development and TMEs
	Single-cell DNA sequencing	Whole genome amplification	Full-genome coverage for detecting chromosomal structural variants; suitable for tumor clonal evolution and pre-implantation genetic diagnosis	High cost and sensitive to low DNA input	Single-cell clonal analysis of tumors; pre-implantation diagnosis of genetic diseases
Targeted sequencing						Focus on regions of interest to reduce sequencing costs; increase sequencing depth for detecting low-frequency mutations	Requires prior knowledge of target regions; technical complexity depends on probe design and capture efficiency	Validation of known cancer mutations in single cells; precision medicine
									Single-cell epigenomics sequencing	Chromatin accessibility sequencing	Antibody-free detection of chromatin openness; infer cell-type-specific transcriptional regulatory networks	Requires thousands of cells; complex data interpretation (needs integration with transcriptomics)	Mechanisms of cell fate determination; transcriptional regulatory network research
DNA methylation sequencing		Resolve single-cell methylation heterogeneity; suitable for stem cell differentiation and cancer epigenomic research	Bisulfite treatment causes DNA loss; high cost and low throughput	Epigenetic regulation studies; cancer epigenomic heterogeneity analysis									

It is a powerful research tool for studying the changes in gene expression and function of cells at the single-cell level. High-throughput sequencing tools have had a profound impact on and made significant contributions to biomedical research, and have pushed the precision and accuracy of tumor molecular biology research to new heights [7].

Sc-seq technology conducts high-throughput gene expression profiling at the single-cell level, enabling in-depth analysis of complex cell populations and characterizing the expression profiles of individual cells. This approach prevents the heterogeneous biological information of individual cells from being masked by the homogenization of a large number of cells [8]. The single-cell transcriptome detection technology has a wide range of applications and is a golden method for studying tumor heterogeneity, immune cells, embryonic development, and other fields. Its core technology involves using microfluidic chips to isolate cells or cell nuclei, ensuring that there is only one cell or cell nucleus in each small reaction system (Gel Bead in Emulsion, GEM). Cells are labeled through Gel Beads with different sequence tags in each GEM, thus enabling the differentiation of cells.

The experimental process generally consists of the following steps: (1) Cell preparation: quality inspection and counting are carried out on the single-cell suspension. The cells that pass the inspection are washed and resuspended to prepare an appropriate cell concentration for machine loading. (2) GEM generation and tagging: samples are loaded according to the expected number of target cells. GEMs are constructed for single-cell isolation. After the normal formation of GEMs, they are collected and reverse-transcribed in a PCR instrument to achieve tagging. (3) Post GEM-RT purification and cDNA amplification: the GEMs are subjected to oil-breaking treatment. Magnetic beads are used to purify and enrich the first-strand cDNA, and then cDNA amplification and quality inspection are carried out. (4) Library construction and quantification: the cDNA that passes the quality inspection is used to construct a second-generation sequencing library. Through experimental processes such as fragmentation, ligation of sequencing adapters, and sample Index PCR, the library is finally quantitatively quality-inspected. (5) Machine sequencing: the constructed library is sequenced using the Illumina HiSeq or NovaSeq platform with the PE150 sequencing mode. After the sequencing data is downloaded, analysis software such as CellRanger, as well as the currently most widely used software in the single-cell field, including Seurat, Monocle, and SingleR, is used for cell filtering, dimensionality reduction and clustering, cell type prediction, differential gene analysis, pseudotemporal trajectory analysis, etc [9,10].

Compared with traditional sequencing, it has two relatively obvious advantages, namely its resolution and heterogeneity. Specifically, single-cell analysis provides higher-resolution measurements than bulk analysis. It allows for the identification and characterization of rare or previously unknown cell types or subtypes within a population, as well as the detection of subtle changes in gene expression or protein levels. Cells within a population can be highly heterogeneous, meaning that different cells may express different genes or have different functions. Single-cell techniques allow for the study of individual cells, providing insights into the heterogeneity of individual cells, which may be overlooked in population-level measurements [11]. Similarly, sc-seq also has certain drawbacks, including the high experimental difficulty of sc-seq technology, the high financial threshold of sc-seq technology, and the small number of pathological specimen sequencing [12,13].

With the continuous maturation of sc-Seq technology, more researchers have applied it to tumor research, revealing the heterogeneity of cells within tumors, depicting the tumor microenvironment (TME), analyzing the mechanisms of tumorigenesis, development and metastasis, screening molecular markers, guiding treatment, evaluating prognosis, and monitoring recurrence. Currently, sc-Seq technology has been widely used in the research of various malignant tumors, such as hematological diseases, breast cancer, lung cancer, liver cancer, colorectal cancer, bladder cancer, and ovarian cancer [14-20].

Research on the application of sc-seq in NAT for esophageal cancer

The treatment modalities for esophageal cancer include surgery, NAT before surgery, perioperative chemotherapy, postoperative chemotherapy, targeted therapy, and immunotherapy. Among them, NAT is playing an increasingly crucial role in the treatment of esophageal cancer [21]. In recent years, due to the advancements in staging methods, treatment regimens, and surgical techniques, the mortality and recurrence rates of patients have decreased to some extent. However, the prognosis of most esophageal cancer patients remains poor [22]. Therefore, it is of great significance to use new technologies to explore the mechanisms of the occurrence and development of esophageal cancer and to identify new targets for its prevention and treatment. Sc-seq technology will serve as a powerful tool in the research of esophageal cancer. It delves into the single-cell level to analyze the heterogeneity between esophageal tumor cells. This technology holds great promise in predicting both the treatment efficacy and prognosis of esophageal cancer patients, establishing a solid foundation for making personalized treatment plans. 

Application of sc-seq in analyzing the TME of esophageal cancer during NAT

The TME is the complex environment where tumor cells grow and develop [23]. TME is composed of tumor cells, infiltrating immune cells, stromal cells, other cell types, and non-cellular tissue components that influence disease progression [24]. Understanding the cell composition of the TME and the dynamic changes in its related transcriptome during tumorigenesis is extremely important for exploring the effectiveness of tumor NAT [25,26]. Analyzing differences in the TME after NAT and investigating the underlying mechanisms can provide a foundation for personalized neoadjuvant treatment of ESCC.

Some research focused on analyzing the tumor tissues of ESCC patients who underwent neoadjuvant chemotherapy and immunotherapy. In this study, single-cell RNA-seq was employed to analyze both these tumor tissues and their matched adjacent normal tissues [27]. The researchers then detected the changes in cell clusters and cellular components within the TME under different treatment regimens. The study found that compared with chemotherapy alone, the combination of immunotherapy and chemotherapy increased the proliferation level of T cells, partially restored the function of exhausted T cells, induced the expansion of specific exhausted CD8+ T cells, increased the production of dendritic cells, and supported the formation of a tumor-immune hot microenvironment. It was also discovered that CD52 and ID3 could serve as biomarkers for ESCC, and high expression of ID3 and CD52 was associated with poor prognosis in ESCC patients. Among the six different fibroblast clusters, CAF2 andCAF5 affected other cancer-associated fibroblast (CAF) clusters through multiple pathways, leading to malignant transformation. Changes in the expression of MHC-I and MHC-II genes in CAF2 and CAF5 were found, which altered the antigen-presenting function of CAFs.

To further explore the changes in fibroblasts within the TME of esophageal cancer, some studies have focused on the alterations of relevant fibroblasts in the TME of ESCC under neoadjuvant chemotherapy combination therapy [28]. It was found that the abundance of the CAFs population changed significantly after neoadjuvant chemotherapy. For example, the infiltration of IL6⁺ and CCL2⁺ immunomodulatory CAF subsets, as well as CD248⁺ mechanoresponsive CAFs, was found to increase. CD248⁺ CAFs impeded CD8⁺ T cell infiltration and drug delivery, while IL6⁺ and CCL2⁺ CAFs contributed to treatment resistance. Interactions between CAFs and T cells were also observed; for instance, the NECTIN2-TIGIT ligand-receptor pair was enriched in treated samples, and TIGIT is a key inhibitory immune checkpoint on T cells [29]. The study identified the functional phenotypes of CAFs associated with poor treatment responses in patients [30].

Application of sc-seq in research on neoadjuvant chemotherapy for esophageal cancer

Neoadjuvant chemotherapy refers to the administration of systemic chemotherapy to patients before esophageal cancer surgery. The aim is to increase the surgical resection rate and reduce the risk of postoperative recurrence and metastasis by down-staging the primary tumor [31]. The combination chemotherapy regimen based on Cisplatin (DDP) and 5-fluorouracil (5-FU) has been widely used globally as part of the treatment plan for esophageal cancer [32]. Relevant studies conclude that neoadjuvant chemotherapy is significantly more effective than surgery alone, improving both the short-term treatment efficacy and long-term survival benefits for patients. Therefore, chemotherapy remains an effective treatment method for most esophageal cancer patients [33].

Through sc-seq of tumor cells from ESCC patients before and after neoadjuvant chemotherapy containing cisplatin [34], the functions of tumor cells are mainly concentrated in aspects such as cell adhesion molecule binding, enzyme inhibitory activity, and cadherin binding. Metabolic analysis mainly focuses on metabolites, degenerative changes, carcinogenesis, and other aspects. Based on the multi-omics analysis of cisplatin-resistant and sensitive cell lines obtained from previous studies, tumor resistance genes were further screened, and a Neoadjuvant Chemotherapy Score (NCS) was constructed [35,36]. It was found that the NCS of tumor cells before neoadjuvant therapy was significantly higher than that after neoadjuvant therapy. Finally, a survival model was constructed to screen out esophageal cancer patients who may clinically benefit from neoadjuvant chemotherapy.

Currently, research on sc-seq in neoadjuvant chemotherapy for esophageal adenocarcinoma is rather limited. To explore the regulatory mechanisms of neoadjuvant chemotherapy from the single-cell perspective of esophageal adenocarcinoma, sc-seq was conducted on four esophageal adenocarcinoma patients who had never received tumor treatment previously and four patients who had received neoadjuvant chemotherapy [37]. The study found that EAC tumors have a complex local microenvironment, which can trigger a strong T-cell immune response, but is restricted by effector cell exhaustion and the expansion of regulatory subsets. Neoadjuvant chemotherapy can reverse these changes. For example, neoadjuvant chemotherapy can regulate the EAC cell population. After treatment, the proportions of NK and proliferative T cells decrease, while the proportions of B cells, endothelial cells, and fibroblasts increase. A differential expression analysis was performed on different cell populations to identify differentially expressed genes (DEGs) after chemotherapy, and it was found that neoadjuvant chemotherapy induced transcriptional changes in different cell populations. This study comprehensively compared the situations before treatment and after neoadjuvant chemotherapy, revealing changes in cell subsets and transcriptional characteristic changes of multiple cell types at the single-cell level, providing a new perspective for understanding the EAC immune response.

Neoadjuvant chemotherapy combined with targeted therapy is a common strategy for treating esophageal malignancies in clinical practice [38,39]. Some researchers performed scRNA-seq on the tumor tissues of a metastatic esophageal cancer patient who received chemotherapy and trastuzumab±pembrolizumab [40]. At the single-cell level, extensive heterogeneity in ERBB2 expression was discovered. In the cell subset with high ERBB2 expression, FGFR3 (a potential biomarker for trastuzumab resistance) was highly expressed. By using paired scRNA-seq data, the clonal composition of tumor biopsies before and after treatment was reconstructed. It was found that most of the clones with high ERBB2 expression were rapidly cleared, but some expanded after treatment, indicating the existence of resistance to trastuzumab and chemotherapy. Gene set analysis showed that highly expressed genes such asMT1H, MT1E, MT2A, and MSMB were associated with trastuzumab resistance. The results of this study contribute to understanding the resistance mechanisms involved in treating HER2+ esophageal cancer, aid in predicting treatment responses and patient prognosis, and provide guidance for optimizing treatment strategies.

Application of sc-seq in NCRT for esophageal cancer

Neoadjuvant chemoradiotherapy (NCRT) means treating tumors comprehensively with both radiotherapy and chemotherapy before surgery [41]. For patients, combining radiotherapy and chemotherapy can wipe out the main tumor lesion and other small lesions. Also, they can enhance each other's effects, thus greatly improving the treatment efficacy [42]. Results from studies like the NEOCRTC5010 study and the CROSS study show that the neoadjuvant concurrent CRT mode gives patients much better survival advantages compared to those who only have simple surgical resection.

The impact of NCRT on the immune microenvironment of ESCC was studied via scRNA-seq. Samples from ESCC patients were analyzed both before and after NCRT [43]. Multiple expression programs of tumor cells prior to treatment were found to be associated with the NCRT response. For example, NCRT augmented the infiltration of CD8+ T cells, yet it also induced their exhaustion. After NCRT, the proportion of total CD8+ T cells increased significantly. Additionally, NCRT influenced the differentiation of CD4+ T cells, altered the state of dendritic cells, enhanced the expression of M2 macrophage markers, and weakened some intercellular interactions. These research results deepen the understanding of the immune response mechanism of ESCC following NCRT. They offer a solid and reliable scientific foundation for the subsequent development of novel immune strategies and their clinical application to improve the treatment outcomes of ESCC. For example, by leveraging immunotherapy based on the PD-L1 pathway, precisely regulating the quantity and activity of regulatory T cells is expected to remarkably enhance the treatment efficacy of NCRT [44].

Application of sc-seq in neoadjuvant immunotherapy for esophageal cancer

The human immune system can recognize and eliminate tumor cells. However, tumor cells can develop certain evasion mechanisms. Immunotherapy aims to treat tumors by activating the body's own immune system and enhancing the ability of immune cells to recognize and kill tumor cells. Immune checkpoint inhibitors(ICIs) represented by programmed death 1(PD-1) in combination with chemotherapy have been approved for first-line neoadjuvant treatment of esophageal cancer [45,46]. Research shows that patients who receive NAT combined with immunotherapy have a higher pathologic complete response (pCR) compared to those who only receive neoadjuvant chemotherapy [47].

Neoadjuvant immunotherapy is usually combined with chemoradiotherapy. Sc-seq was carried out on ESCC patients who received postoperative neoadjuvant treatment with partial pathological response and those who underwent pure surgery [48]. Among them, Ep-C2 type cells (a tumor cell subset with strong antioxidant characteristics) had the highest survival proportion in patients treated with the combination of chemoradiotherapy and immunotherapy. Their marker genes, such as OSGIN1 and CYP4F3, were related to the oxidative stress response and drug metabolism pathways. Transcription factor regulatory network analysis showed thatMAFG and NFE2L2, the key factors in the antioxidant response signaling pathway, were activated. This indicates that the antioxidant response has a potential tolerance effect on chemoradiotherapy combined with immunotherapy. In the previous single-cell transcriptome sequencing cohort of ESCC treated with pure surgery, the Ep-C2 cell subset was also observed to exist before neoadjuvant treatment, proving that the inherent Ep-C2 cell subset with antioxidant response evolved into a dominant tolerant population after chemoradiotherapy combined with immunotherapy. Based on the gene characteristics of Ep-C2, developing potential drugs to inhibit the activity of Ep-C2 cells may improve the efficacy of chemoradiotherapy combined with immunotherapy.

To reveal the potential regulatory network among immune cells and the dynamics of functional clones, sc-seq and other analyses were carried out on samples from ESCC patients before and after neoadjuvant immunotherapy combined with chemotherapy [49]. They found that GZMK+ effector memory T cells were enriched in responders. Additionally, they elaborated on the exhaustion and memory transition mechanisms of myeloid cells from the perspectives of antigen presentation and co-stimulation. Through bulk-RNA analysis, they discovered that S100A7 was positively correlated with the proportions of CXCL13+ exhausted T cells and TNFRSF9+ regulatory T cells, demonstrating that* S100A7* is associated with the immunosuppressive TME [50]. By using techniques such as scRNA and T/B cell receptor sequencing and integrating various data for analysis, they focused on the dynamic changes and interactions of immune cells after neoadjuvant immunotherapy combined with chemotherapy, providing a new perspective for the mechanism research of neoadjuvant immunotherapy combined with chemotherapy for ESCC.

## Conclusions

In conclusion, research on sc-seq within various neoadjuvant treatment regimens for esophageal cancer presents significant potential. Currently, sc-seq still has numerous drawbacks, including but not limited to high sample quality requirements, high cost, lack of sequencing information, and difficulty in reproducibility. With the advancement of sc-seq technology, these disadvantages will gradually be solved. The results of cell sequencing will be more accurate, and the sequencing process will be simpler. The first problem that needs to be solved is how to translate the achievements of sc-seq technology into clinical applications. It requires the full cooperation of basic and clinical research. Hopefully, more clinical trials in the future will bring exploration and optimization of neoadjuvant treatment decisions. It is predicted that in the future, sc-seq technology can be used to detect new therapeutic targets for esophageal cancer patients. It can help make personalized neoadjuvant treatment plans for them and save the lives of more esophageal cancer patients.
